# Resolution of Left Bundle Branch Block After Calcium Administration in the Prehospital Setting

**DOI:** 10.7759/cureus.32442

**Published:** 2022-12-12

**Authors:** Phillip Bonar, Marshall A Frank

**Affiliations:** 1 Emergency Medicine, Florida State University College of Medicine, Sarasota, USA; 2 Emergency Medicine and EMS Medicine, Florida State University College of Medicine, Sarasota, USA

**Keywords:** prehospital setting, intravenous calcium, hyperkalemia induced ekg changes, bundle branch block, hyperkalemia

## Abstract

Hyperkalemia is a medical emergency with potentially severe consequences that can be avoided by early recognition and effective treatment. Electrocardiogram (ECG) changes can help elucidate hyperkalemia prior to obtaining lab results and assist in early decisions on treatment, especially in the prehospital setting. ECG changes commonly associated with hyperkalemia are peaked T-waves, PR prolongation, P-wave flattening, QRS widening, or a sine-wave pattern at severely elevated potassium levels. Bundle branch blocks (BBBs) are associated with hyperkalemia but are less common and less well known in this setting. We report a case of a prehospital ECG showing a left bundle branch block (LBBB) in a patient who had end-stage renal disease, and the prehospital treatment with calcium chloride lead to resolution of the LBBB. The patient was eventually found to have a serum potassium level of 6.1 mEq/L.

## Introduction

Hyperkalemia is a common electrolyte derangement that can have severe consequences such as cardiac conduction abnormalities. The level at which hyperkalemia is likely to cause these severe manifestations is usually above 7.0 mEq/L; however, this cutoff value is not especially sensitive or specific [1]. The death rate for severe hyperkalemia not treated rapidly has been reported to be as high as 67% [2]. ECG changes can help elucidate hyperkalemia prior to obtaining lab results and assist with early decisions on treatment. Early ECG changes more commonly associated with hyperkalemia are peaked T-waves and QT shortening, followed by PR prolongation, P-wave flattening, and QRS widening, with the ominous “sine-wave” occurring later, with very severe hyperkalemia. Bundle branch blocks (BBBs) are associated with hyperkalemia but are less common and less well known in this setting [3]. ECG changes in hyperkalemia represent an indication for emergent treatment. This should start with a membrane-stabilizing agent such as calcium. The next goal should be to reduce the serum potassium level by driving it into the cell, such as insulin, albuterol, and bicarbonate. Finally, more definitive treatment involves clearing potassium from the body with medications, such as diuretics or gastrointestinal tract potassium binders, or with hemodialysis [4].

## Case presentation

**Figure 1:**
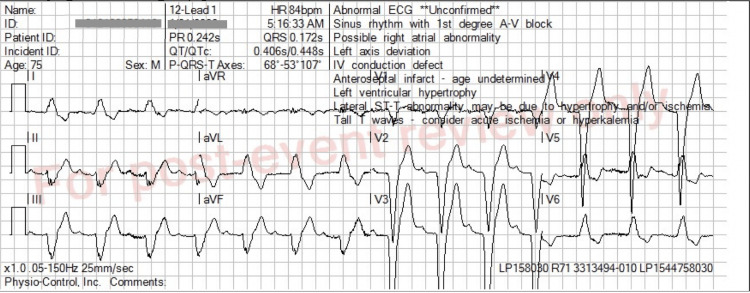
Initial ECG First-degree AV block with a PR interval of 242 ms, LBBB pattern with wide QRS complexes at 172 ms, normal discordant ST-T changes, and tall, peaked T waves. ECG, electrocardiogram; LBBB, left bundle branch block; AV, atrioventricular

**Figure 2:**
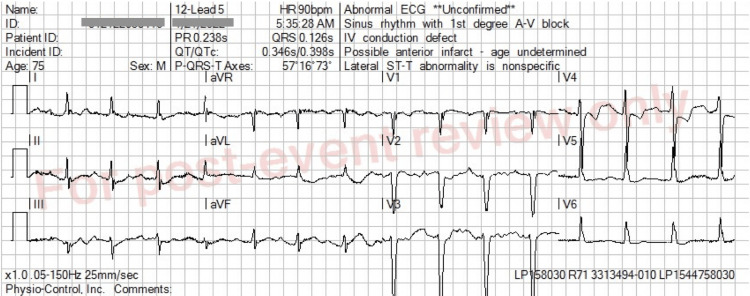
ECG after 1 gram of intravenous calcium chloride given QRS duration is significantly decreased, now borderline LBBB is at 126 ms, and T waves are significantly shorter. PR interval also mildly decreased to 238 ms. ECG, electrocardiogram; LBBB, left bundle branch block

Emergency medical services (EMS) received a call for a 75-year-old male with dyspnea. On arrival, they found the man sitting upright, saying that he could not breathe. He revealed he had a history of congestive heart failure and end-stage renal disease (ESRD) on hemodialysis, with his last dialysis two days ago. There were bibasilar audible rales on examination. Peripheral oxygen saturation was 70% on room air; he was placed on nasal cannula oxygen and transitioned to continuous positive airway pressure (CPAP). Oxygen saturations improved to 86-88% on CPAP. Blood pressure (BP) was initially 190/108. He was given two sublingual nitroglycerin 0.4 mg followed by an additional third 0.4 mg dose with BP decreasing to 131/75. A 12-lead ECG was performed, and was noted in the prehospital report to show “peaked T-waves” (Figure 1). The case was then discussed between this EMS provider and an EMS captain in another vehicle regarding decision to give calcium in this situation. A joint decision was made to administer 1 gram of calcium chloride intravenously. Subsequent ECGs showed improvement in the size of the T-waves and overall appearance of the ECG. On review of the ECGs for this case report, the initial ECG pattern was noted to be a left bundle branch block (LBBB) with peaked T waves that did resolve after administration of calcium chloride (Figures 1, 2). The patient was later confirmed at the receiving hospital emergency department to have a potassium level of 6.1 mEq/L.

## Discussion

Hyperkalemia is a common electrolyte abnormality that is typically seen in acute or chronic renal failure and is often associated with polypharmacy. Medications that are frequently thought to contribute include angiotensin-converting enzyme inhibitors, angiotensin II receptor blockers, potassium-sparing diuretics, NSAIDs, and beta-blockers, among others [5]. Other comorbidities common in the elderly such as coronary artery disease (CAD), hypertension (HTN), and diabetes mellitus can also contribute to decreased ability of the body to regulate potassium [6,7].

ECG changes in hyperkalemia are a useful tool in initial evaluation, as they provide evidence for the need for emergent treatment, and do so more quickly than a blood potassium level can be obtained. Common ECG changes associated with hyperkalemia are peaked T waves, flattened P waves, widening of the QRS, or “sine wave” pattern when very severe. Serum potassium levels are thought to be generally associated with specific ECG changes. Usually, peaked T waves are the initial finding as the potassium level reaches 5.5-6.5 mEq/L. At levels of 6.5-8.0 mEq/L, ECG findings such as P wave flattening, prolonged PR interval, and QRS widening are seen. As the potassium level rises over 8.0 mEq/L, findings such as absence of P wave, BBBs or other conduction delays, significant widening of the QRS, the classic sine wave pattern, ventricular fibrillation, pulseless electrical activity, or asystole can be seen [3]. However, it is important to note that specific ECG changes in hyperkalemia are neither sensitive nor specific to the presence or the severity of hyperkalemia. In fact, 50-64% of patients with potassium levels > .5 mEq/L present with no ECG changes [8].

LBBB is a common abnormal ECG finding. It is usually caused by chronic stress on the cardiac conducting system leading to degeneration. Conducting system stress leading to LBBB can be caused by various chronic cardiovascular conditions such as HTN, cardiomyopathy, or CAD. Acute cardiac conditions can also lead to significant conducting system damage and LBBB. Examples of this scenario include acute MI, myocarditis, endocarditis, and cardiac surgery. Acute hyperkalemia is a less common cause of LBBB. The mechanism behind the development of an LBBB secondary to hyperkalemia is thought to be depressed intracardiac conduction secondary to changes in the cardiac membrane potential [9].

It is reasonable to suspect our patient had chronic HTN, some degree of cardiomyopathy, and potentially multiple other reasons to have an LBBB besides hyperkalemia alone. Also, in retrospect, his potassium level was 6.1 mEq/L, less than expected to cause a BBB (potassium > 8.0 mEq/L) [3]. These factors made it somewhat unexpected that the LBBB resolved after treatment for hyperkalemia. It was not known at the time if the patient had a preexisting LBBB. The response to treatment supports the fact that specific ECG changes are not reliable predictors of the potassium level. The ECG did appear to have tall, peaked T waves as well, which aided in the prehospital recognition and treatment of hyperkalemia.

## Conclusions

Hyperkalemia is a relatively common medical emergency that requires rapid identification and treatment. The ECG is an important tool for early identification of hyperkalemia in the appropriate clinical setting. This is especially important in the prehospital setting. We demonstrate in this case a unique scenario in which the decision was made in the prehospital setting to treat a patient with a history of ESRD and findings of LBBB on ECG with IV calcium chloride, which lead to resolution of the LBBB.
